# Supplementary material for: Factors influencing self-management in relation to type 2 diabetes in Africa: A qualitative systematic review
Source: PLoS One. 2020 Oct 22;15(10):e0240938. doi: 10.1371/journal.pone.0240938 (PMC7580976; doi:10.1371/journal.pone.0240938)
Supplement: S1 File — (DOCX) [file pone.0240938.s001.docx]

S1 File: Systematic Review Protocol. Factors Influencing Self-management in relation to Type 2 Diabetes in Africa

**Introduction**

Diabetes is a chronic metabolic condition of public health concern and one of the top four non-communicable diseases (NCDs) on the agenda of the United Nations (UN). Thus, Member States as part of the Sustainable Development Goals (SDGs) have set a target of reducing premature deaths due to NCDs by one third by 2030 [1]. This target calls for strategic policy intervention and proactive service delivery. A recent estimate by the International Diabetes Federation (IDF) indicates that approximately 425 million people (8.8%) between the ages of 20 -79 years have diabetes, the majority from lower- and middle-income countries [2]. Africa has recorded an increasingly high prevalence of the disease. For instance, according to IDF [3] in 2015, 14 million people had diabetes in Africa and this figure is projected to rise to 34 million by 2040. This suggests that diabetes has reached epidemic levels in all parts of Africa. For example, in a meta-analysis, Hilawe et al [4] estimated diabetes prevalence to be 5.7% in Sub-Saharan Africa. Another population-based study of diabetes prevalence in African countries from 1980-2014 indicated that, diabetes in women and men had increased from 4.1% to 8.9% and 3.4% to 8.5% respectively [5]. According to IDF [2] in 2017, diabetes accounted for 298,160 deaths (6% of all mortality) in Africa. These alarming figures put pressure on the health systems of developing African nations who still experience the highest global prevalence of NCDs, including HIV [6], Tuberculosis and Malaria [7].

Key elements in the management and prevention of diabetes and its complications involve life style modifications in areas such as exercise, diet, medication adherence, blood glucose monitoring and foot care, collectively referred to as ‘self-management activities’ [8, 9, 10]. Research in high income settings shows that following a structured diabetes self-management (DSM) approach leads to better clinical and quality of life outcomes [11]. In order to understand the key factors that influence DSM and thus to develop appropriate self-management models for sub-Saharan African countries, it is essential to understand peoples’ views and experiences of managing their condition.

**Aim**

. To synthesise qualitative evidence that investigated the views and experiences of persons with type 2 diabetes in Africa regarding diabetes self-management (DSM). Specific objectives of the review are:

- To explore and describe the self-management experiences of persons living with type 2 diabetes in Africa.
- To describe self-management behaviours (exercise, medication adherence, diet, blood glucose monitoring and foot care) of people with diabetes
- To identify from persons with diabetes own perspective, factors that act as barriers and facilitators of self-management of type 2 diabetes.

**Materials and Methods**

This qualitative systematic review will be reported following the ENTREQ guidelines [12].

**Search strategy**

A comprehensive search strategy will be constructed using key words and MesH headings in five databases: MEDLINE, CINAHL, EMBASE, PsyINFO and Scopus. The databases will be searched from January 2000 to December 31^st^, 2019. In addition, hand-searches of the reference lists of all included studies will be undertaken

**Inclusion and exclusion criteria**

Table 1 gives details of the inclusion and exclusion criteria will be applied to the review. The WHO definition for ‘Africa Region’ will guide the selection of studies for inclusion

**Table 1: Inclusion and exclusion criteria**

| Variable | Inclusion | Exclusion |
| --- | --- | --- |

| Population (Participants) | - persons with type 2 diabetes - Adults above 18 years of age | - Type 1 diabetes persons - Gestational diabetes - Participants below age 18 years - Non-diabetic persons |
| --- | --- | --- |

| Phenomenon of interest | - Views and experiences of persons with type 2 diabetes (beliefs, perceptions, attitudes, understanding, behaviours) regarding self-management of diabetes (exercise, diet, blood glucose monitoring, medication adherence and foot care) | - Research that investigated other aspects of persons with type 2 diabetes than their views and experiences regarding self-management |
| --- | --- | --- |
| Context | - ‘WHO Africa Region’ - Study setting includes homes or community settings and hospitals | - Studies conducted outside the ‘WHO Africa Region’ |
| Study Design | - Qualitative study of any design and the qualitative findings of mixed methods studies | - Other (non-qualitative) study designs |
| Language | - English | - Studies in other languages |

**Quality assessment**

Study quality will be independently assessed by two reviewers using the Joanna Briggs Institute Qualitative Assessment and Review Instrument (JBI-QARI) [13].

**Data extraction**

The JBI-QARI tool for data extraction will be used to extract key study characteristics (methodology, methods, settings, geographical context, participants, phenomenon of interest, data analysis method and researcher’s conclusions). The findings and discussion sections of the papers will be extracted and coded to develop the synthesis. This process will be undertaken by two reviewers. and any discrepancies in interpretation will be discussed.

**Synthesis**

Synthesis of findings will be done in three stages as outlined by Thomas and Harden [14]: (i) systematic coding of the results of individual studies; (ii) grouping of codes together based on similarity in meaning or shared characteristics to form descriptive themes, and, (iii) interpretation of higher order analytical themes.

**Ethics and dissemination**

**This will be a systematic review. Therefore, ethical approval will not be required since there will be no human beings involvement.**

**Review Registration number** CRD42018102255.

References

1. United Nations. Transforming our world: the 2030 Agenda for Sustainable Development [Available from: [https://sustainabledevelopment.un.org/post2015/transformingourworld](https://sustainabledevelopment.un.org/post2015/transformingourworld%20)
2. International Diabetes Federation. Diabetes Atlas 8th Edition 2017 [Available from: <http://www.diabetesatlas.org/resources/2017-atlas.html>.
3. International Diabetes Federation. IDF Diabetes Atlas 7^th^ edition [updated 2015. Available from: <https://www.idf.org/e-library/epidemiology-research/diabetes-atlas/13-diabetes-atlas-seventh-edition.html>
4. Hilawe EH, Yatsuya H, Kawaguchi L, Aoyama A. Differences by sex in the prevalence of diabetes mellitus, impaired fasting glycaemia and impaired glucose tolerance in sub-Saharan Africa: a systematic review and meta-analysis. Bulletin of the World Health Organization. 2013;91:671-82D.
5. NCD Risk Factor Collaboration (NCD-RisC) – Africa Working Group Trends in obesity and diabetes across Africa from 1980 to 2014: an analysis of pooled population-based studies. International Journal of Epidemiology. 2017;46:1421–1432
6. UNAIDS. 2010 Report on the global AIDS epidemic. 2010, Geneva: UNAIDS [Available from: <https://www.unaids.org/globalreport/documents/20101123_GlobalReport_full_en.pdf>
7. World Health Organization. Global tuberculosis control: WHO report 2010 [Available from: <https://apps.who.int/iris/handle/10665/44425>
8. Bagnasco A, Di Giacomo P, Da Rin Della Mora R, Catania G, Turci C, Rocco G, et al. Factors influencing self‐management in patients with type 2 diabetes: a quantitative systematic review protocol. Journal of advanced nursing. 2014;70(1):187-200.
9. Jordan DN, Jordan JL. Self-care behaviors of Filipino-American adults with type 2 diabetes mellitus. Journal of Diabetes and its Complications. 2010;24(4):250-8.
10. Preumont V, Hermans M, Buysschaert M, Jamart J. Special considerations in the management and education of older persons with diabetes. Diabetes & Metabolism. 2009;35:A60.
11. Brewer-Lowry AN, Arcury TA, Bell RA, Quandt SA. Differentiating approaches to diabetes self-management of multi-ethnic rural older adults at the extremes of glycemic control. The Gerontologist. 2010;50(5):657-67.
12. Tong A, Flemming K, McInnes E, Oliver S, Craig J. Enhancing transparency in reporting the synthesis of qualitative research: ENTREQ. BMC medical research methodology. 2012;12(1):181.
13. Joana Briggs Instittute. Joanna Briggs Institute Reviewers’ Manual 2014 Edition [Available from <http://joannabriggs.org/assets/docs/sumari/reviewersmanual-2014.pdf>.
14. Thomas J, Harden A. Methods for the thematic synthesis of qualitative research in systematic reviews. BMC medical research methodology. 2008;8(1):45.
