# Supplementary material for: Factors influencing self-management in relation to type 2 diabetes in Africa: A qualitative systematic review
Source: PLoS One. 2020 Oct 22;15(10):e0240938. doi: 10.1371/journal.pone.0240938 (PMC7580976; doi:10.1371/journal.pone.0240938)
Supplement: S2 File — (DOCX) [file pone.0240938.s002.docx]

**S2 File: Search Strategy**

| **Key words or concepts** | **Other related terms or synonyms** |
| --- | --- |
| Diabetes Mellitus | Diabetes mellitus, type 2, Diabetes, Type II diabetes, non? Insulin dependent diabetes |
| Views and experiences | View*, experience*, understand*, comprehend*, concern*, opinion*, perspective*, belie*, perception* |
| Self-management | Self-management, self-care, exercise*, physical activit*, diet, food and nutrition, medication*, treatment*, foot care, blood glucose monitor* |
| Africa | African countries, developing countries, low income countries, resource limited countries, resource limited setting*, non? Western countries |

*truncation

**Search strategy (MEDLINE) and results**

| Search #ID | Search options | Results |
| --- | --- | --- |
| 1 | Diabetes Mellitus/ or Diabetes Mellitus, Type 2/ or diabetes mellitus, type 2.mp. | 219772 |
| 2 | type II diabetes.mp. | 7355 |
| 3 | type ii diabetes mellitus.mp. | 2425 |
| 4 | 1 or 2 or 3 | 223035 |
| 5 | View*.mp | 413717 |
| 6 | Experience*.mp | 909731 |
| 7 | Understand*.mp | 929874 |
| 8 | Comprehen*.mp | 289958 |
| 9 | Concern*.mp | 516807 |
| 10 | Opinion*.mp | 101978 |
| 11 | Perspective*.mp | 255782 |
| 12 | Belie*.mp | 256720 |
| 13 | Perception* | 377696 |
| 14 | 5 or 6 or 7 or 8 or 9 or 10 or 11 or 12 or 13 | 3385457 |
| 15 | self-management.mp. or Self-Management/ | 14453 |
| 16 | self-care.mp. or Self Care/ | 38845 |
| 17 | exercise*.mp. | 327192 |
| 18 | physical activit*.mp. | 89964 |
| 19 | diet.mp. or diet/ or "diet, food, and nutrition"/ | 409376 |
| 20 | medication adherence/ or medication*.mp | 292117 |
| 21 | treatment*.mp. or "treatment adherence and compliance"/ | 4359847 |
| 22 | Diabetic Foot/ or foot care.mp. | 8299 |
| 23 | Blood Glucose Self-Monitoring/ or blood glucose monitor*.mp. | 6712 |
| 24 | 15 or 16 or 17 or 18 or 19 or 20 or 21 or 22 or 23 | 5131225 |
| 25 | Africa.mp. or AFRICA/ | 136370 |
| 26 | African countries.mp | 5927 |
| 27 | developing countries.mp. or Developing Countries/ | 111487 |
| 28 | low income countries.mp. | 4601 |
| 29 | resource limited setting*.mp. | 4391 |
| 30 | non? western countries.mp. | 298 |
| 31 | 25 or 26 or 27 or 28 or 29 or 30 | 237395 |
| 32 | 4 AND 14 AND 24 AND 31 | 243 |

*truncation
