# Supplementary material for: Factors influencing self-management in relation to type 2 diabetes in Africa: A qualitative systematic review
Source: PLoS One. 2020 Oct 22;15(10):e0240938. doi: 10.1371/journal.pone.0240938 (PMC7580976; doi:10.1371/journal.pone.0240938)
Supplement: S3 File — (DOCX) [file pone.0240938.s003.docx]

**S3 File.** **List of Countries covered by WHO African Region**

| Algeria  Angola  Benin  Botswana  Burkina Faso  Burundi  Cameroon  Cabo Verde  Central African Republic  Chad  Comoros  Congo  Côte d'Ivoire  Democratic Republic of the Congo  Equatorial Guinea  Eritrea | Ethiopia  Gabon  Gambia  Ghana  Guinea  Guinea-Bissau  Kenya  Lesotho  Liberia  Madagascar  Malawi  Mali  Mauritania  Mauritius  Mozambique  Namibia | Niger  Nigeria  Rwanda  Sao Tome and Principe  Senegal  Seychelles  Sierra Leone  South Africa  South Sudan  Swaziland  Togo  Uganda  United Republic of Tanzania  Zambia  Zimbabwe |
| --- | --- | --- |
