# Supplementary material for: Factors influencing self-management in relation to type 2 diabetes in Africa: A qualitative systematic review
Source: PLoS One. 2020 Oct 22;15(10):e0240938. doi: 10.1371/journal.pone.0240938 (PMC7580976; doi:10.1371/journal.pone.0240938)
Supplement: S4 File — (DOCX) [file pone.0240938.s004.docx]

**S4 File:** Quality appraisal of studies using JBI-QARI instrument

| **Study Reference** | **Q1** | **Q2** | **Q3** | **Q4** | **Q5** | **Q6** | **Q7** | **Q8** | **Q9** | **Q10** |
| --- | --- | --- | --- | --- | --- | --- | --- | --- | --- | --- |
| Abdulrehman et al. (2015) | Y | Y | Y | Y | Y | Y | N | Y | Y | Y |
| BeLue., Diaw and Ndao (2013) | Y | Y | Y | Y | Y | N | N | Y | U | U |
| O’Briem, von Rooney and Ricks 2015) | Y | Y | Y | Y | Y | N | N | Y | Y | Y |
| Mendenhall and Norris (2015) | U | Y | Y | Y | Y | U | U | Y | Y | Y |
| Adeniyi et al. (2015) | U | Y | Y | Y | Y | N | N | Y | Y | Y |
| Matwa et al. (2003) | U | Y | Y | Y | Y | Y | N | Y | Y | Y |
| Tewahido and Berharne (2017) | U | Y | Y | Y | Y | N | N | Y | Y | Y |
| Hjelm and Mufunda (2010) | U | Y | Y | Y | Y | N | U | Y | Y | Y |
| Steyi and Phillips (2014) | Y | Y | Y | Y | Y | N | N | Y | Y | Y |
| Hjelm and Beebwa (2013) | Y | Y | Y | Y | Y | Y | N | Y | Y | Y |
| Hjelm and Nambozi (2008) | U | Y | Y | Y | Y | Y | N | Y | Y | Y |
| Awah et al (2008) | Y | Y | Y | Y | Y | N | N | Y | Y | Y |
| Doherty et al (2014) | U | Y | Y | Y | Y | U | U | Y | Y | Y |
| de-Graft Aikins (2003) | U | Y | Y | Y | Y | U | U | Y | Y | Y |
| de-Graft Aikins (2005) | Y | Y | Y | Y | Y | N | U | Y | Y | Y |
| de-Graft Aikins (2015) | U | Y | Y | Y | Y | N | N | Y | Y | Y |

Y – yes; N – no; U – unclear; Q – question number on JBI-QARI instrument
