# Supplementary material for: Factors influencing self-management in relation to type 2 diabetes in Africa: A qualitative systematic review
Source: PLoS One. 2020 Oct 22;15(10):e0240938. doi: 10.1371/journal.pone.0240938 (PMC7580976; doi:10.1371/journal.pone.0240938)
Supplement: S5 File — (DOCX) [file pone.0240938.s005.docx]

**S5 File: Data Extracted template**

| Paper Title:  Reviewers:  Authors. Year:  Journal: Record number:  Study description |
| --- |
| METHODOLOGY: |
| METHODS: |
| PHENOMENON OF INTEREST: |
| SETTING: |
| GEOGRAPHICAL: |
| CULTURAL: |
| PARTICIPANTS: |
| DATA ANALYSIS: |
| AUTHORS CONCLUSION: |
|  |

| **Findings** | **Illustration from publication (page numbers)** | **Evidence** | | |
| --- | --- | --- | --- | --- |
|  |  | **Unequivocal** | **Credible** | **Unsupported** |
|  |  |  |  |  |
|  |  |  |  |  |
|  |  |  |  |  |
|  |  |  |  |  |

Extraction of findings complete: Yes No
