# Supplementary material for: Factors influencing self-management in relation to type 2 diabetes in Africa: A qualitative systematic review
Source: PLoS One. 2020 Oct 22;15(10):e0240938. doi: 10.1371/journal.pone.0240938 (PMC7580976; doi:10.1371/journal.pone.0240938)
Supplement: Attachment — Submitted filename: Response to Reviewers.docx [file pone.0240938.s008.docx]

**Response to Reviewer(s) PONE-D-20-14887R1**

Factors Influencing Self-management in relation to Type 2 Diabetes in Africa: A Qualitative Systematic Review

PLOS ONE

Dear Editor,

Thank you for reviewing our manuscript and the points raised. We have revised the manuscript; taking into consideration PLOS ONE’s publication criteria and comments raised by the Academic Editor.

The table below presents a detail response to the reviewers’ comments

Authors’ Response to Reviewer’s Comments

| **Editor’s Comments** | **Authors Response** |
| --- | --- |
| Further discussion on future directions, clinical implications and practical recommendations for improving self-management in Africa. | Thank you for these points raised. Within the discussion section of the revised paper, we have added in substantive further points regarding the clinical implications of our findings, whilst also giving additional recommendations for interventions that could be developed and evaluated to improve self-management.  To make our recommendations for policy and practice clearer, we have also summarised these in tables 4 and 5 |
